# Mouse models of deep vein thrombosis

**DOI:** 10.1007/s00772-016-0227-6

**Published:** 2016-12-12

**Authors:** T. Schönfelder, S. Jäckel, P. Wenzel

**Affiliations:** 1 Centrum für Thrombose und Hämostase, Universitätsmedizin Mainz, Mainz, Germany; 2 Medizinische Klinik, Universitätsmedizin, Johannes-Gutenberg-Universität Mainz, Langenbeckstr. 1, 55131 Mainz, Germany

**Keywords:** Venous thromboembolism, Ultrasonography, Optical imaging, In vivo imaging, Experimental animal model, Venöse Thromboembolie, Ultraschall, Optische Bildgebung, In-vivo-Bildgebung, Experimentelles Tiermodell

## Abstract

The pathogenesis of venous thromboembolism (VTE) is still not completely understood. Experimental animals in which human deep vein thrombosis can be modeled are useful tools to investigate the pathogenesis of VTE. Besides the availability of transgenic and genetically modified mice, the use of high frequency ultrasound and intravital microscopy plays an important role in identifying thrombotic processes in mouse models. In this article, an overview about the application of various new technologies and existing mouse models is provided, and the impact of venous side branches on deep vein thrombosis in the mouse model is discussed.

## Zusammenfassung

Noch immer ist die Pathogenese des venösen Thromboembolismus (VTE) nicht bis ins Detail geklärt. Experimentaltiere, in denen eine tiefe Venenthrombose generiert wird, sind hilfreich bei der Erforschung der VTE-Pathogenese. Neben der Verfügbarkeit von transgenen bzw. genetisch modifizierten Mäusen spielt die Verwendung von Hochfrequenzultraschall und Intravitalmikroskopie bei der Darstellung thrombotischer Prozesse im Mausmodell eine große Rolle. Der Beitrag gibt einen Überblick über die Verwendung neuer Technologien sowie über bestehende Modelle und stellt den Einfluss von venösen Seitenästen auf die tiefe Venenthrombose im Mausmodell dar.

Approximately 40,000 people die annually in Germany from venous thromboembolism, which is usually the result of deep vein thrombosis [12]. Due to the mild or lack of symptoms of the underlying disease, diagnosis is often too late or not recognized at all. Further investigation of predisposing factors is imperative, and research on appropriate animal models is important to better understand this disease.

Deep vein thrombosis and resulting pulmonary embolism in the sense of venous thromboembolism (VTE) are among the most common diseases worldwide and are associated with a high mortality rate [11]. Along with myocardial infarction and stroke, VTE is a common cardiovascular disease with an annual incidence within the U.S. Caucasian population of 108 per 100,000 [11]. Many risk factors (e. g., age, sex, immobilization after surgery, trauma, and cancer) have been identified [10]. Nevertheless predisposing factors and the exact pathogenesis are still not completely understood. Attempts to further define various aspects of this disease often reach the limits of in vitro and ex vivo diagnostics because multiple factors influence this complex vascular disease. Thus, the interactions between the vein wall, inflammatory cells, platelets, and coagulation factors play a significant role in the pathogenesis of venous thrombosis. The possibility of using transgenic mouse strains is, therefore, an important argument to perform studies on VTE using the animal model. Deletion and overexpression in the mouse genome and depletion of various inflammatory cell types have significantly contributed to the understanding of the thrombotic processes. Thus, using a mouse model of venous thrombosis, Brühl et al. [20] were able to demonstrate the essential influence of neutrophils and monocytes in the initial thrombus formation. This finding would not have been possible without studies in the mouse model. Effective methods must therefore

- be able to ideally model the human disease,
- contribute to animal protection aspects by keeping the number of experimental animals to be examined to a minimum, and
- optimize existing models – also in terms of their comparability.

## Venous thrombosis in a mouse model

Thrombotic processes can be observed in a murine small vein model, for example, in mesenteric veins, or in large veins of the animal model. To induce venous thrombosis, the inferior vena cava is mainly used. Studies in small vessels, such as the mesenteric veins, are usually designed to analyze acute thrombogenetic processes, while the inferior vena cava thrombosis model can be used to analyze both acute and chronic stages [5]. The size of this vein also offers the possibility to gather more material for analytical purposes in order to determine various parameters, such as thrombus size and composition, or inflammatory status of the vein wall.

The use of various mouse knockout strains in experimental animal models has still further advantages with respect to comparability with the human organism. Thus, some parallels arise from the species comparison, including the initial infiltration of inflammatory neutrophils and monocytes, fibrin and collagen production in thrombus formation, and clot retraction and new infiltrating vascular channel formation in thrombus tissue during thrombus resolution [8].

**Fig. 1:**
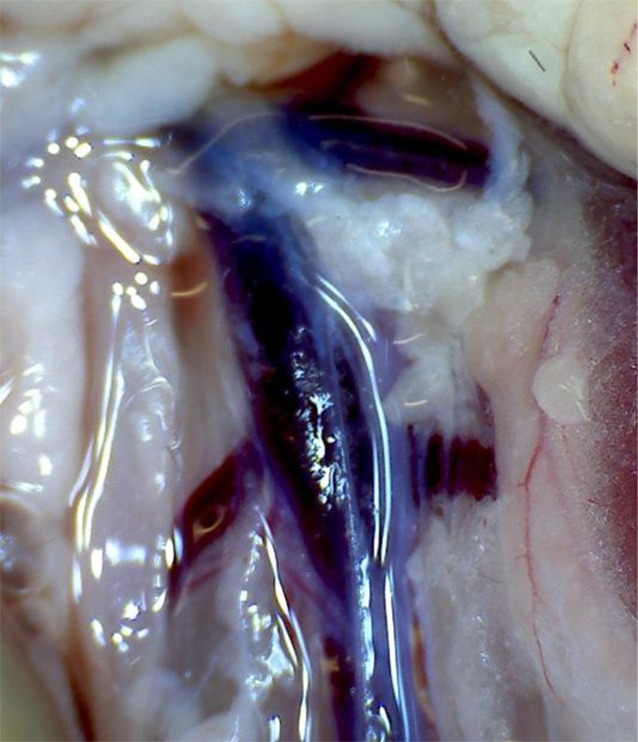
The inferior vena cava is the most commonly used vessel in the venous thrombosis model. The segment caudal to the left renal vein is used. Anatomical variations in the number of side branches in this area are common

Currently, there are various approaches to induce thrombus formation in the inferior vena cava. To access the surgical site, a midline laparotomy is performed initially under deep anesthesia. After exteriorization of the bowel to the side, the inferior vena cava can be visualized. The section to be used is located caudal to the renal vein and extends to the bifurcation of the two iliolumbar veins (Fig. 1). The 5 most commonly used models are briefly outlined below.

### Induction by ferric chloride and photochemically with Rose Bengal

A small piece of filter paper saturated with a 3.5% solution of ferric chloride is directly applied to the exposed vein and removed after 2–3 min. The oxidative damage induced to the vessel wall results in the formation of an occlusive thrombus within a matter of minutes. The rate of thrombus initiation and the size of the resulting thrombus can be influenced by varying concentration (10%/20% ferric chloride) and exposure time.

Another method is the injection of photoreactive substances (e. g., Rose Bengal), which accumulate in the lipid bilayer of endothelial cells. Irradiation with light initiates a photochemical reaction that leads to the formation of oxygen radicals, thus, resulting in damage to the endothelium [7].

These models are mainly used to study acute thrombogenesis. Because of the toxic or redox chemical stimulus, these models do not ideally represent the pathophysiology of the majority of clinically occurring deep vein thromboses [13].

### Inferior vena cava stasis or ligation model

Stasis in a vessel is achieved by complete ligation of the inferior vena cava in the region caudal to the renal vein. The side branches opening laterally into the vena cava are additionally ligated and partially posterior branches cauterized. This model reflects complete occlusion of the vessel in the clinical scenario. The thrombus size is relatively constant; however, the endothelial damage resulting from total ligation is not negligible. This model can also be used to study both acute and chronic processes; the focus is more on observation of chronic stages with the analysis of recanalization and thrombus resolution.

### Induction by electrolytic stimulation

A silver-coated copper wire attached to a 25-gauge needle is inserted into the subcutaneous tissue and into the caudal inferior vena cava. Application of direct current (250 μA) over about 15 min promotes the formation of free radicals, which results in endothelial cell activation [6]. Lateral venous side branches of the inferior vena cava are ligated in this area. The circulation is not affected, and there is minimal endothelial injury at the needle puncture site. Compared with the IVC (inferior vena cava) stasis model, the thrombus grows with the blood flow, and total occlusion of the vessel does not occur. Compared with other models, both acute and chronic stages of venous thrombosis can be studied. Possible limiting factors for this model are equipment and time costs.

### Inferior vena cava stenosis model

**Fig. 2:**
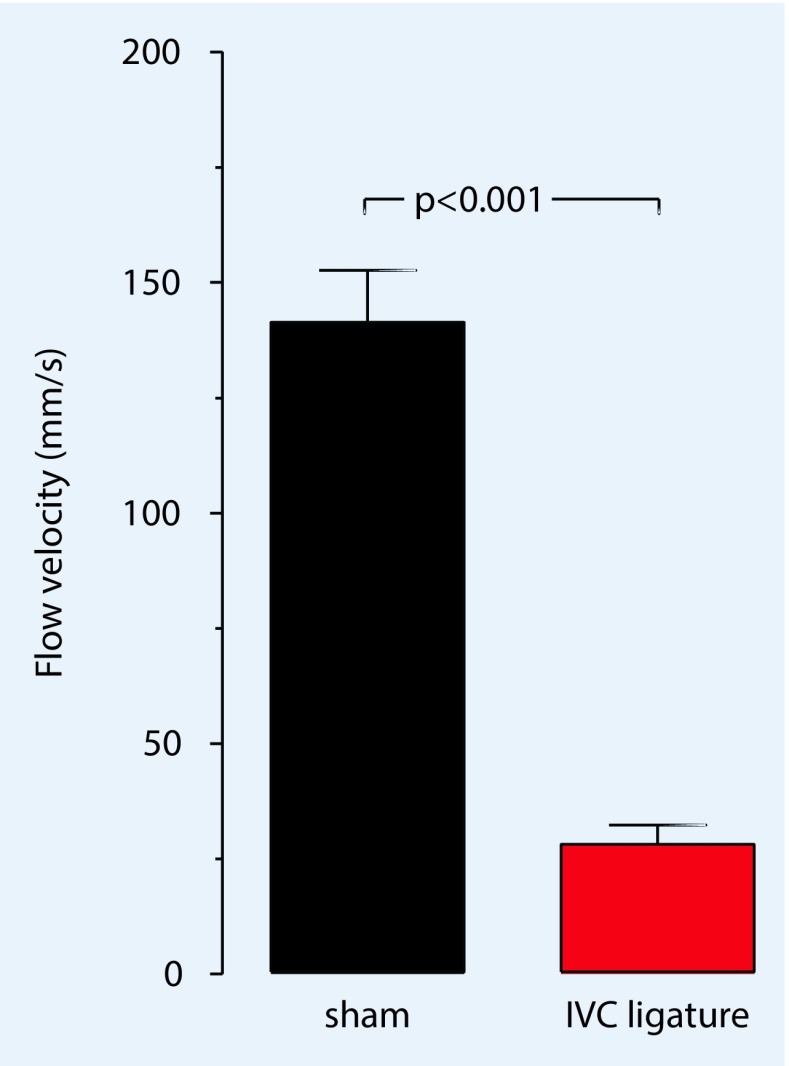
For the formation of a venous thrombosis in the IVC (inferior vena cava) stenosis model, a reduction of the original flow by approximately 80% is necessary. In Fig. 5, the average pulsed-wave Doppler flow velocity (mm/s) in the vena cava before and after stenosis of the vessel is shown. (Adapted from [3]; with kind permission from IOS, Amsterdam)

As in the stasis model, ligation caudal to the renal veins is performed in the inferior vena cava (IVC) stenosis model; however, only an object (e. g., a needle) is used to create the stenosis and is removed again during the course of the procedure, thus, a decrease in blood flow of approximately 80% is guaranteed (Fig. 2). Brühl et al. [20] reported finding no endothelial damage in the area of the ligation.

**Fig. 3:**
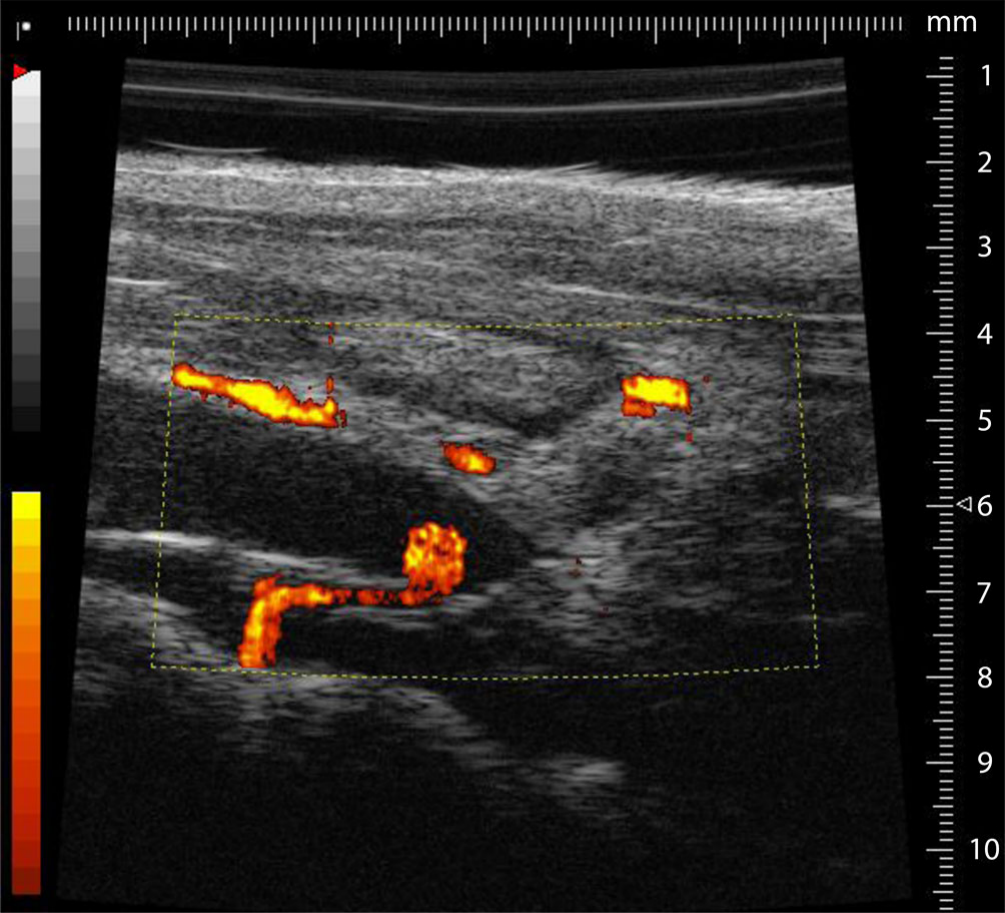
Quantitative representation of the flow rate in the longitudinal section of the inferior vena cava after ligation. The colored areas indicate the inflow region of a dorsal side branch of the inferior vena cava. Due to its proximity to the ligature, there is no thrombus formation

In the literature, various modifications of this model are described, including the use of vascular clips which result in not only a reduction in flow (Fig. 3) but also in endothelial damage [5]. Furthermore, in this model, there are modifications concerning the lateral branches of the vena cava. Some authors completely ligate the visible branches lying lateral to the inferior vena and leave the posterior branches untouched. The purpose of this modification is a more constant thrombus formation. A serious disadvantage is the large variation in thrombus size. In addition, some mice do not develop a thrombus even after successful surgery.

In summary, vena cava ligature with induction of stenosis without any modifications represents the best model for deep vein thrombosis in human disease. There is only a decrease of the flow rate, the endothelium remains intact, and the thrombus forms under existing circulation.

## Imaging methods

### Intravital video fluorescence microscopy

The technique of intravital video fluorescence microscopy allows the real time visualization of cellular processes in vivo. By using fluorescent dyes in vivo or ex vivo that are well-tolerated by the animal, different cells and cellular components can be visualized in the living animal by fluorescence microscopy. In most cases, the resulting image data are recorded using a fluorescence light microscope with a water-immersion objective in combination with a digital camera and sent to a computer where the entire data analysis is performed with software. The microscope used is equipped with specific filters that are compatible with the fluorescent dyes. Nucleated blood cells are often labeled in vivo with various DNA markers (e. g., acridine orange, SYTO dyes), while the platelet and microparticle fractions are isolated first from the blood of a donor animal and are transfused into the recipient animal after ex vivo staining with dichlorofluorescein or rhodamine B [20]. Cell-specific antibodies that are labeled with fluoro-chromes selectively bind to platelets [9], leukocytes [20], fibrinogen [2], and fibrin [16, 18] can be visualized without affecting cell function. Antibodies and fluorescent dyes are usually administered via jugular vein catheter or by the tail vein.

**Fig. 4:**
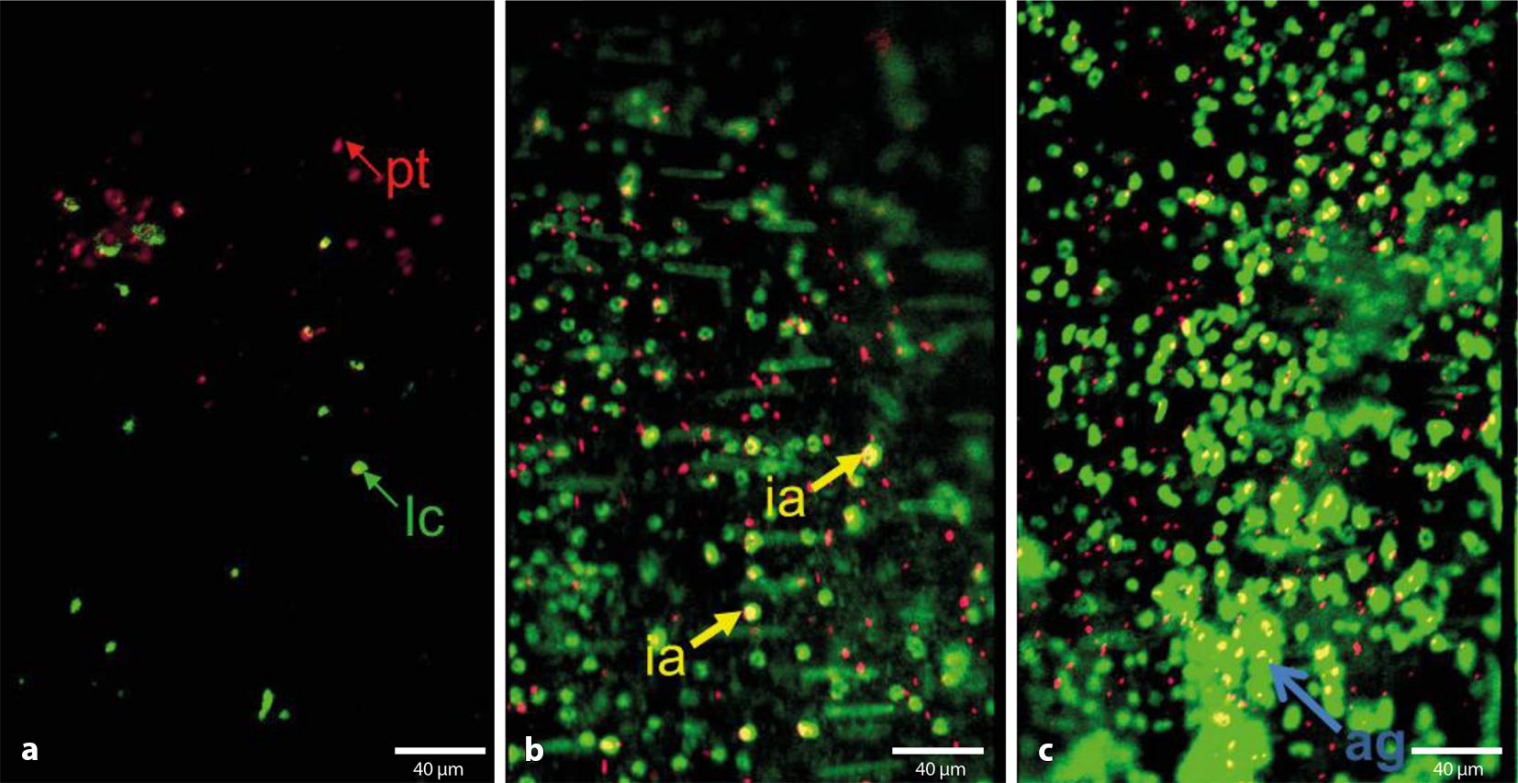
Imaging with a dual view system using intravital epifluorescence microscopy at various time points after stenosis. Dual view of platelets stained with rhodamin B and leukocytes stained with acridine orange. Platelets (*pt*) *red*, leukocytes (*lc*) *green*, platelet–leukocyte interactions (*ia*) *yellow *and aggregate formation (*ag*). **a** 1 h, **b **3 h, **c **6 h after stenosis

Intravital microscopic examination of the various venous thrombosis models is performed immediately after induction of thrombosis and depending on the model usually not longer than 6 h on deeply anesthetized animals under strict control of anesthesia. Thus, especially in the IVC stenosis model, the initial phase of venous thrombus formation can be examined in the area between the sinister renal vein and the common iliac veins, as well as cell–cell and cell–vessel wall interactions (e. g., adherent, rolling cells, aggregation surface) and cell velocities (frame-to-frame method) can be detected and analyzed [20]. Using dual-view systems or two digital cameras, beams can be separated and different spectra of the fluoro-chromes can be simultaneously recorded. With this method, it is possible to obtain interactions of different components involved in thrombosis (Fig. 4).

Using the IVC stenosis model in combination with intravital video fluorescence microscopy, the kinetics of leukocyte subpopulations of monocytes and neutrophils, and the role of NETs (neutrophil extracellular traps) in the early phase of venous thrombus formation could be detected [20]. Further developments in video fluorescence microscopy include confocal in vivo imaging and 2‑photon microscopy. Advantages of 2‑photon microscopy include the depth of penetration into the tissue, image quality, and the possibility to visualize protein structures (e. g., collagen fibers) using the second harmonic signal [4, 15].

### High-frequency ultrasound

Using high-frequency ultrasound, e. g., with the VisualSonics Vevo 770® High-Resolution Imaging System (Visual-Sonics, Toronto, Canada; [1, 3]), the on-set and progression of venous thrombosis in a mouse model can be monitored. Ultrasound transducers with a frequency of 40 MHz, which have better spatial and temporal resolution, are a suitable tool for in vivo imaging studies in small mammals.

**Fig. 5:**
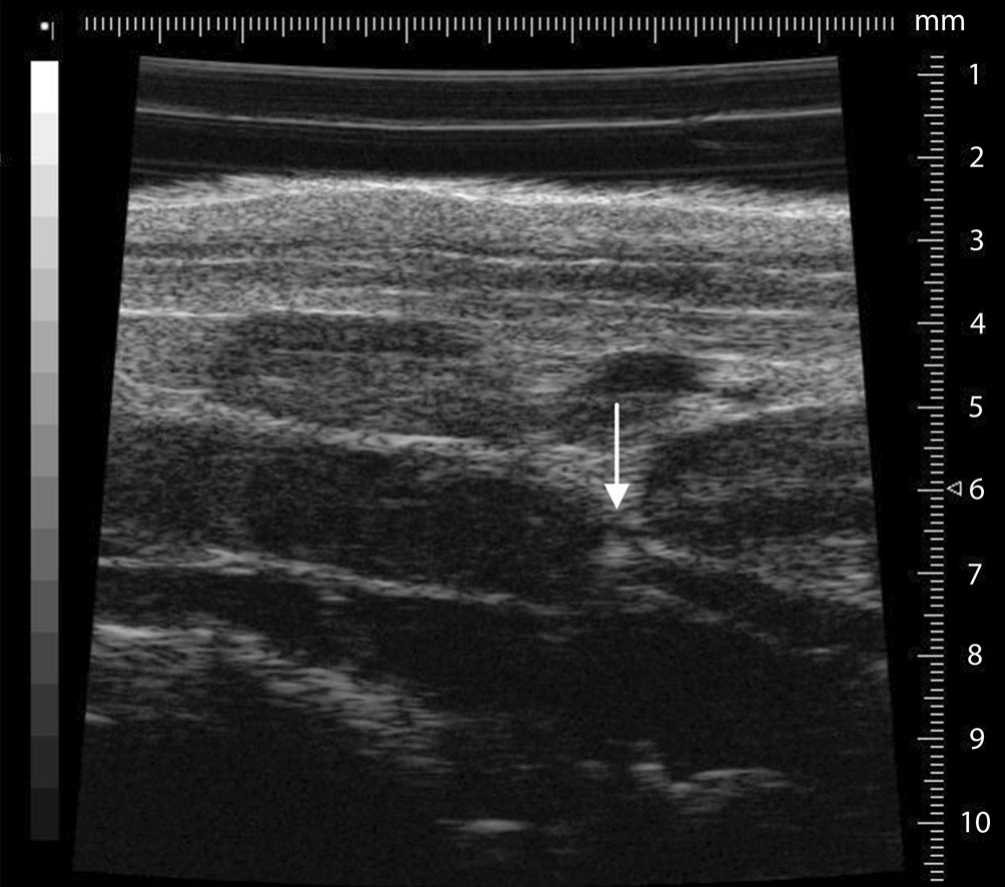
The long-axis B‑mode view provides a good view of the area of the inferior vena cava, including ligature (*arrow*) and already formed thrombus (*left *of the ligature)

Studying mice is much more complicated than studying humans. Thus, it is necessary to perform the ultrasound under isoflurane anesthesia (0.5–1.05% with 0.05–0.1 l/min 100% O_2_). In addition, a fixation unit which can be heated and can record an electrocardiogram is needed. The area to be imaged must be completely free from fur prior to the study. To prevent excessive cooling of the animal, the ultrasound gel should be warmed to 37 °C prior to use. It is imperative that the bodytemperature be measured using a rectal probe during the period of investigation. First, the long-axis view provides a view of the ligated section (Fig. 5); the aorta may initially be helpful to identify the desired section. The pulsed-wave (pw) Doppler mode can be used to display the movement of the corpuscular components in blood, their direction, and flow rate. Using pw-Doppler, it is possible to distinguish between two vessels that are in close vicinity to another. A further advantage is the power Doppler mode, which can provide information about the movement of the corpuscular components of the blood (Doppler signal) by means of color representation in the B‑mode view (Fig. 2). This function is particularly suitable for quantitative representation of slow flow rates and provides information on vascularity. In the analysis and presentation of thrombotic processes, the increase in blood flow in the vessel can be presented quantitatively.

**Fig. 6:**
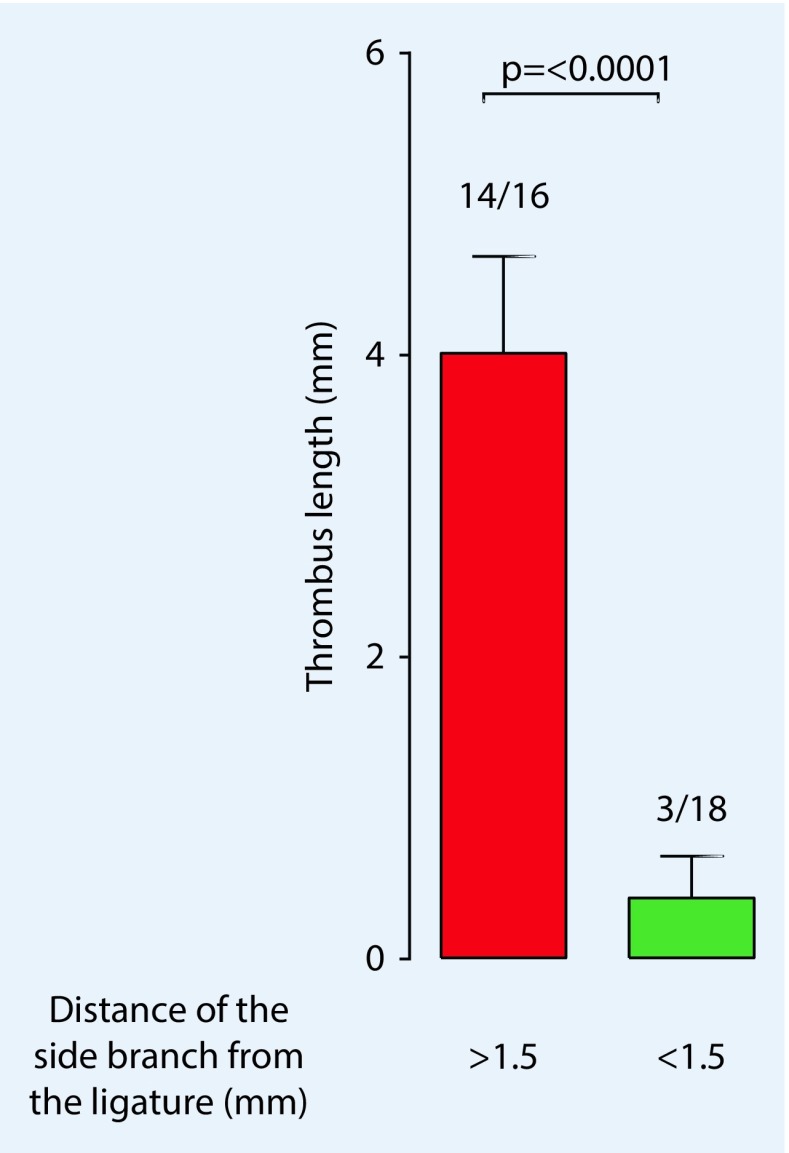
Sonographically determined values show that thrombus length is significantly influenced (*p* < 0.0001) by dorsal side branches of the vena cava when they are located within 1.5 mm from the ligature. (Adapted from [3], with kind permission from IOS, Amsterdam)

Aghourian et al. [1] demonstrated the possibilities of using high-frequency ultrasound to monitor thrombus formation in the stasis and ferric chloride models. The high correlation of sonographically determined thrombus length and the values measured after thrombus resection demonstrated the possibility of a new, noninvasive method that allows the development and progression of thrombosis to be monitored. This was also confirmed for the stenosis model by our group in the following year and we were also able to show the influence of the side branches (Fig. 6) on thrombus formation, which cannot be ignored when using this model [3].

## Influence of venous side branches on the development of VTE

The vascular anatomy in mice is highly variable, particularly the number of vessels opening into the vena cava. Often there are one or two lateral branches and approximately the same number of dorsal branches that are poorly visible during the preparation. As described above in association with the thrombosis models, there are several ways to deal with these vessels. However, complete ligation of the side branches almost inevitably results in nonspecific endothelial damage, which can have an immediate and uncontrollable influence on thrombus formation.

The aim of the work presented here was to determine how strongly these side branches opening into the vena cava influence the IVC stenosis model. The first goal was to sonographically clarify how the opening of the side branches into the vena cava affects thrombus length. In the literature, it is mentioned in association with the stenosis model that thrombus formation ends at the opening of the side branches into the vena cava [5]. After analyzing our data, we found a correlation between the opening of the posterior lateral branches with open (“patent”) flow and the thrombus length. Interestingly, however, the flow rate where the side branch opens into the vena cava does not appear to have an influence on the formation or the resulting thrombus length.

However, in our measurements, we were able to demonstrate a particular phenomenon: if the opening of the side branch into the vena cava is within 1.5 mm of the ligature, thrombus formation is prevented. Diaz et al. [5] had already described the disadvantage of this model: even after successful surgery, thrombus formation does not occur in some mice. As sonographic examination shows, the opening of a side branch in the area around the ligature seems to be the cause (Fig. 6).

As mentioned above, there are some modification options when using the VC stenosis model; thus, the hypothesis was to test whether ligation of visible vessels also ensures a more constant thrombus formation or a more constant thrombus length.

Using high-frequency ultrasound (Vevo 770), each mouse was tested 48 h after successful surgery. Thrombus size and the distance between the opening of the side branches from the long axis were measured. Furthermore, flow velocity in the side branch was determined using pw-Doppler.

The analysis of our results showed no association between ligation of the lateral side branches and a more constant thrombus length or thrombus formation in general. The variation in size and formation process remained unchanged, which is probably due to the dorsal side branches that were not ligated.

Although we identified side branches as being determinants in thrombus formation (at least in the mouse model), the role of venous side branches and their influence on the events of deep vein thrombosis remains insufficiently understood. In humans, collateral vessels appear to represent natural ways around obstructions [17]. Another aspect is that the pockets of the venous valves are attributed to an increased risk potential associated with the development of deep vein thrombosis. Precisely in these locations, the different flow rates and hypoxic condition that exist can promote thrombus formation [14]. Somewhat underrepresented in experimental research are altered shear stress rates, hematological changes, or hyperviscosity that occur in the context of VTE [19].

## Conclusion

- High-frequency ultrasound is a noninvasive method that is particularly useful to study venous thrombosis. Thus, the formation and the course of venous thrombosis can be followed in various IVC thrombosis models.
- The influence of venous side branches, particularly the dorsal branches, on thrombus formation should always be considered when using the vena cava stenosis model in order to interpret the results correctly.
- In addition to our results, it must be taken into consideration that some factors such as the sex and age of the animals, which are not modifiable in the experiments [20], have an influence on thrombus formation.
